# Experimental Primary Brain Calcification Model and Its Application to Pathogenesis Mechanism Analysis and Therapeutic Research

**DOI:** 10.3390/neurolint18010004

**Published:** 2025-12-24

**Authors:** Hisaka Kurita, Junya Murata, Kazuki Ohuchi, Yuichi Hayashi, Masatoshi Inden

**Affiliations:** 1 Laboratory of Medical Therapeutics and Molecular Therapeutics, Gifu Pharmaceutical University, 1-25-4 Daigaku-nishi, Gifu 501-1196, Japan; kurita@gifu-pu.ac.jp (H.K.); 233004@gifu-pu.ac.jp (J.M.); ohuchi-ka@gifu-pu.ac.jp (K.O.); y-hayashi@tsuruga-nu.ac.jp (Y.H.); 2 Department of Clinical Pathophysiology and Functional Morphology, Faculty of Nursing Science, Tsuruga Nursing University, 78-2-1 Kizaki, Tsuruga-city, Fukui 914-0814, Japan

**Keywords:** Primary Brain Calcification, PBC models, iPS cells

## Abstract

Primary Brain Calcification (PBC) is a neurodegenerative disorder of unknown etiology that results in bilateral calcifications within the brain. PBC symptoms vary, including Parkinsonian symptoms and psychiatric symptoms. Abnormalities in phosphate metabolism within the brain are hypothesized to be a mechanism underlying the onset of PBC, but the precise pathophysiological mechanism remains unclear. Furthermore, no fundamental treatment or therapeutic agent for PBC has been established. Previous studies have reported *SLC20A2*, *PDGFB*, *PDGFRB*, *XPR1*, *MYORG*, *JAM2*, *CMPK2*, and *NAA60* as causative genes for familial PBC. Elucidating the pathophysiological mechanisms of PBC and developing treatments and therapeutic agents requires appropriate experimental disease models. Knockout mice and mutant mice targeting familial causative genes have been reported to be useful as in vivo models of PBC. Furthermore, several disease-specific iPS cells for PBC have been reported, suggesting their potential utility as PBC models. This paper reviews each familial causative gene and current PBC models, including genetically modified animals and disease-specific iPS cells, and examines their usefulness for understanding disease mechanisms and advancing therapeutic research.

## 1. Introduction

Primary Brain Calcification (PBC) is an idiopathic neurodegenerative disorder causing bilateral calcifications within the brain. The symptoms of PBC vary widely and include Parkinsonian symptoms, psychiatric symptoms, and others. Abnormalities in phosphate metabolism within the brain are thought to be a mechanism underlying the onset of PBC, but the precise pathophysiological mechanism remains unclear. Therefore, no established treatments or medications exist for PBC. Previous studies have reported *SLC20A2*, *PDGFB*, *PDGFRB*, *XPR1*, *MYORG*, *JAM2*, *CMPK2*, and *NAA60* as causative genes for familial PBC. SLC20A2 is a phosphate transporter that facilitates the uptake of inorganic phosphate (Pi) into cells [1]. PDGFB is a growth factor involved in angiogenesis and cell proliferation [2]. PDGFRB is the receptor tyrosine kinase for PDGFB and is involved in cell proliferation [3]. XPR1 is a phosphate transporter that transports Pi out of cells [4]. MYORG could possess α-galactosidase activity [5,6]. JAM2 has been reported as a protein involved in tight junctions [7]. CMPK2 has been reported to be involved in nucleotide phosphorylation [8]. NAA60 has been reported to possess N-terminal acetyltransferase activity [9]. Although various causative genes for PBC have been reported, elucidating the pathophysiological mechanisms of PBC and developing treatments and therapeutic drugs requires appropriate experimental disease models based on these causative genes. Knockout mice and mutant mice targeting familial causative genes have been reported to be useful as in vivo models of PBC. Furthermore, several reports have described disease-specific iPS cells (iPSC) for PBC, which could also prove useful as PBC models. This paper reviews each familial causative gene and current PBC models, including genetically modified animals and disease-specific iPS cells, examining their pathophysiological mechanisms and utility for therapeutic research.

## 2. Genes Causing Familial PBC

Several familial causative genes for PBC have been identified (Table 1) [10,11]. Analysis of these familial causative genes is crucial for developing disease models of PBC. Below is an overview of these familial causative genes.

### 2.1. SLC20A2

*SLC20A2* is the first identified causative gene for PBC and encodes the Pit2 protein, one of the sodium-dependent type III phosphate transporters that transport Pi into cells [1]. SLC20A2 is expressed in a wide range of organs, but it has been reported to be particularly highly expressed in the globus pallidus, thalamus, and cerebellum of the brain [12,13]. The *SLC20A2* mutation follows an autosomal dominant inheritance pattern [1]. The *SLC20A2* gene is the most common causative gene for PBC, with *SLC20A2* mutations identified in approximately 58% of genetic-confirmed PBC patients [10]. In *Slc20a2* homozygous knockout (KO) mice and autopsies of *SLC20A2* mutant PBC patients, calcification has been reported to be observed both outside the cerebral blood vessels and within the vascular walls [14,15,16,17]. In *Slc20a2* homozygous KO mice, calcification was particularly observed within the pericyte and astrocyte cells [15]. It has been suggested that functional abnormalities caused by calcification of these pericytes and astrocytes could lead to permeability issues in the blood–brain barrier (BBB) [18]. A recent study using cell-specific *Slc20a2* KO mice suggested that astrocytic *Slc20a2* could play a crucial role in pathological conditions associated with PBC, such as brain calcification [19]. Furthermore, Pit2 is known to be expressed on the apical membrane of rat choroid plexus epithelial cells, suggesting that Pit2 plays a crucial role in the active transport of Pi from cerebrospinal fluid (CSF) to blood to maintain Pi homeostasis in CSF [20]. Pi concentrations in the CSF of PBC patients carrying the *SLC20A2* mutation and in *Slc20a2* homozygous KO mice were elevated [15,21,22]. In PBC patients with the *SLC20A2* mutation, a localized hyperphosphatemic environment in the CSF is considered one cause of calcification.

### 2.2. PDGFB

*PDGFB* was reported as a familial causative gene for PBC in 2013 [23]. PDGFB is a growth factor associated with angiogenesis and cell proliferation [2]. PDGFB is expressed in nerve cells and brain vascular endothelium [23]. PDGFB forms homodimers or heterodimers with PDGFA and acts as a ligand for PDGFRA or PDGFRB [24]. PDGFB gene mutations follow an autosomal dominant inheritance pattern, and heterozygous mutations account for approximately 13% of genetic-confirmed PBC patients [10]. *Pdgfb*-deficient mice and vascular endothelial cell-specific *Pdgfb* knockout mice exhibit cerebral calcification and BBB disruption [23,25]. Therefore, PDGFB dysfunction-induced breakdown of the BBB is thought to trigger intracerebral calcification.

### 2.3. PDGFRB

*PDGFRB* was reported as a familial causative gene for PBC in 2013 [3]. *PDGFRB* gene mutations are autosomal dominant, and heterozygous mutations account for approximately 4% of genetic-confirmed PBC patients [10]. PDGFRB is one of the receptor tyrosine kinases, and its ligands are PDGFB, PDGFC, and PDGFD [24]. PDGFRB is expressed in vascular smooth muscle cells (SCMs) and pericytes [26,27]. PDGFRB signaling is crucial for the proliferation and migration of vascular smooth muscle cells and pericytes, and is deeply involved in the formation of the blood–brain barrier [3,28]. Similarly to PDGFB mentioned above, dysfunction of PDGFRB is thought to cause breakdown of the BBB and is considered one factor contributing to intracerebral calcification.

### 2.4. XPR1

*XPR1* was reported as a familial causative gene for PBC in 2015 [4]. The *XPR1* gene mutation is autosomal dominant, and heterozygous mutations account for approximately 6% of genetic-confirmed PBC patients [10]. XPR1 is a phosphate transporter that transports Pi from the intracellular to the extracellular [4]. XPR1 dysfunction is thought to increase intracellular Pi accumulation and cause calcification [4]. Interestingly, it has been reported that intracellular Pi uptake mediated by Pit2 increases PP-IP via IP6K, and that the SPX domain of XPR1 senses this PP-IP to promote phosphorus efflux [29]. Based on the above, XPR1 maintains intracellular Pi homeostasis in coordination with Pit2, and disruption of this process is thought to be involved in the pathogenesis of PBC.

### 2.5. MYORG

*MYORG* was reported as a familial causative gene for PBC in 2018 [5]. The *MYORG* gene mutation is an autosomal recessive inheritance, and the homozygous mutation accounts for approximately 14% of genetic-confirmed PBC patients [10]. Based on its amino acid sequence, MYORG could possess α-galactosidase activity [5,6]. MYORG has been reported to be highly expressed in the cerebellum, particularly in the endoplasmic reticulum and astrocytes [5]. MYORG dysfunction could likely suppress protein glycosylation and contribute to the pathogenesis of PBC. Interestingly, it has been reported that MYORG is involved in the distribution of Pit2 to the cell membrane in astrocytes [19]. The molecular mechanisms underlying the pathophysiology of MYORG-related PBC remain unclear, and further research should be required, including the identification of MYORG-interacting proteins.

### 2.6. JAM2

*JAM2* was reported as a familial causative gene for PBC in 2020 [7]. The *JAM2* gene mutations follow an autosomal recessive inheritance pattern, and homozygous mutations account for approximately 3% of genetic-confirmed PBC patients [10]. JAM2 is a protein involved in tight junctions and is thought to play a crucial role in cell polarity, endothelial permeability, and the function of the BBB [7,30]. JAM2 is highly expressed in the caudate nucleus of the brain. Furthermore, JAM2 is highly expressed in endothelial cells and astrocytes [30]. Dysfunction of JAM2 leads to loss of intercellular adhesion, causing impaired solute permeability and potentially contributing to cerebral calcification.

### 2.7. CMPK2

*CMPK2* was reported in 2022 as an autosomal recessive familial causative gene for PBC [31]. CMPK2 belongs to the nucleoside monophosphate kinase family and is involved in the salvage pathway for the phosphorylation of dCMP, dUMP, CMP, and UMP in mitochondria [32]. CMPK2 is highly expressed in the hippocampus and cerebellum of the brain. It is also expressed in neurons and vascular endothelial cells [31]. Dysfunction of CMPK2 could also affect mitochondrial function and could be associated with brain calcification.

### 2.8. NAA60

*NAA60* was reported in 2024 as an autosomal recessive PBC familial causative gene [33], and homozygous mutants account for approximately 2% of genetic-confirmed PBC patients [10]. NAA60 acetylates the N-terminus of various membrane proteins and localizes to the Golgi apparatus [34]. Interestingly, it has been reported that the N-terminus of Pit2 protein could be acetylated, and loss of NAA60 reduces Pit2 expression levels on the cell membrane [33]. Further research is needed to clarify the role of NAA60 in the molecular mechanisms underlying PBC pathology.

## 3. PBC Models

Disease models of PBC are crucial for developing treatments and therapies for PBC. Generally, KO mice with mutations in familial causative genes or loss-of-function mutations, as well as disease-specific iPSC, are considered useful models. The following provides an overview of the current situation of PBC models.

### 3.1. In Vivo Models

Several genetically modified mice targeting the familial causative genes of PBC have been reported (Table 2). Among these, *Slc20a2* KO mice are well studied. Of these, many reports are based on studies involving C57BL/6NTac-*Slc20a2^tm1a-(EUCOMM)Wtsi/Ieg^* mice (EM: 05549) [14,15,18,21,35,36,37,38]. Multiple reports have established that in this mouse model, significant intracerebral calcification is observed primarily in the midbrain and thalamus by approximately one year of age at the latest in homozygous KO mice [35,37,38]. Additionally, elevated Pi levels in cerebrospinal fluid have been reported in these homozygous KO mice [21,36]. Similarly, elevated Pi levels in cerebrospinal fluid have been reported in PBC patients harboring the *SLC20A2* mutation [22]. Furthermore, a characteristic phenotype is that homozygous mice are born with smaller body weight and skeletal structure, and tend to have lower survival rates [15,36]. This growth inhibition and reduced survival rate phenotype may also be associated with placental calcification in homozygous KO mice [35]. Furthermore, BBB disruption [18] and spatial learning memory impairments and sensorimotor gating deficits were also confirmed in homozygous KO mice [38]. We also generated Slc20a2 KO mice similar to the previously reported C57BL/6NTac-*Slc20a2^tm1a-(EUCOMM)Wtsi/Ieg^* mice and similarly observed brain calcification in 11-month-old homozygous mice. Furthermore, weight loss and reduced survival rates were also confirmed [17]. In recent years, astrocyte-specific *Slc20a2* KO mice and genetically modified mice expressing human *SLC20A2* mutations have been generated. Phenotypes in these models also show cerebral calcification and elevated Pi levels in CSF [19,39].

There are few reports using genetically modified animals based on other PBC causative genes. In *Pdgfb* gene-deficient mice, including *Pdgfb*^ret/ret^ exhibiting functional deficiency and *Pdgfb*^−/−; R26P+/0^ mice with *Pdgfb* expression restricted to vascular endothelium, cerebral calcification and BBB disruption are observed [23,25]. Knockout mice for *Xpr1* also exist, but homozygous KO mice are lethal due to severe growth retardation caused by placental and embryonic calcification [40]. Therefore, *Xpr1* heterozygous KO mice were used for analysis. While cerebral calcification was observed, unlike *Slc20a2* KO mice, the Pi concentration in CSF decreased [41]. Homozygous KO mice for the *Myorg* gene exhibit brain calcification [5] and elevated Pi levels in CSF [19], similar to *Slc20a2* KO mice. The *Cmpk2* homozygous KO mice or knock-in mice showed calcification of thalamus, and disruption of mitochondrial function [31]. On the other hand, *Jam2* KO mice show no brain calcification, but histological vacuoles in the brain and gait abnormalities are observed [7]. Based on the above, *Slc20a2* KO mice appear to be a suitable PBC model at this time, as they exhibit brain calcification, elevated CSF Pi levels, and abnormalities in higher brain functions. Regarding genetically modified mice targeting other PBC causative genes, further phenotypic analysis should be necessary to utilize them as PBC models. A knock-in model using the CRISPR/Cas9 system in a PBC model mouse targeting SLC20A2 has been reported [39]. We believe it is important to expand the development of CRISPR knock-in model PBC models for other causative genes as well. Since organ-specific calcification is observed in PBC, in vivo PBC models are considered essential for studying disease mechanisms and therapeutic drug research.

**Table 2:** Summary of In Vivo/in vitro PBC models.

Resourse Name	Target Gene	Information of Gene Modification or Variant	Brain Calcification	Other Phenotypes	Reference
C57BL/6NTac-Slc20a2*^tm1a-(EUCOMM)Wtsi/Ieg^* mice (EM: 05549)	Slc20a2	Slc20a2 gene knockout	Calcifications were found in basal ganglia, cortex, and thalamus in homozygous 19-week-old mice (Jensen et al., *J Mol Neurosci*, 2013 [14]). Calcifications were found in the brains of heterozygous and homozygous 1-year-old mice (Wallingford et al., *Brain Pathol*, 2017 [36]) Calcifications were found in the hypothalamus, midbrain, thalamus and pons in homozygous 20-week-old mice (Jensen et al., *Am J Pathol*, 2018 [15]). Calcifications were found in the brains of homozygous 3-, 6-, and 12-month-old mice (Nahar et al., *Brain Pathol*, 2020 [37]) Calcifications were found in midbrain and hypothalamus in homozygous 80-day-old mice, and ventral striatum, basal forebrain, hypothalamus, thalamus, midbrain, and pons in 10-month-old homozygous mice (Ren et al., *Front Genet*, 2021 [38]).	Pi concentration of CSF was increased in homozygous 3-week-old mice (Jensen, Autzen, and Pedersen, *Neurogenetics*, 2016 [21]). Placental calcifications were found in heterozygous dam (Wallingford, Gammill, and Giachelli. *Reprod Biol*, 2016 [35]). Fetal growth retardation was found in hetrozygous and homozygous embryos at embryonic day 17.5 (Wallingford, Gammill, and Giachelli. *Reprod Biol*, 2016 [35]). Pi concentration of CSF was increased in homozygous 1-year-old mice (Wallingford et al., *Brain Pathol*, 2017 [36]). Body weight was reduced in homozygous 6-week-old mice (Wallingford et al., *Brain Pathol*, 2017 [36]). Hydrocephalus and premature death were observed in homozygous mice (Wallingford et al., *Brain Pathol*, 2017 [36]). Both microphthalmia and cataracts were observed in homozygous 6-week-old mice (Wallingford et al., *Brain Pathol*, 2017 [34]). Body weight was reduced in homozygous mice (Jensen et al., *Am J Pathol*, 2018 [14]). Congenital and global developmental delay, lean body mass, skeletal malformation, and a high proportion of unilateral or bilateral eye defects were found in homozygous mice (Ren et al., *Front Genet*, 2021 [36]) Spatial learning memory impairments and sensorimotor gating deficits were observed in homozygous 8-month-old mice (Ren et al., *Front Genet*, 2021 [36]). Impairment of blood–brain barrier (BBB) permeability was observed in homozygous mice (Zhang et al., *Front Mol Neurosci*, 2023 [18]).	Jensen et al., *J Mol Neurosci*, 2013 [14] Jensen, Autzen, and Pedersen, *Neurogenetics*, 2016 [21] Wallingford, Gammill, and Giachelli. *Reprod Biol*, 2016 [35] Wallingford et al., *Brain Pathol*, 2017 [36] Jensen et al., *Am J Pathol*, 2018 [15] Nahar et al., *Brain Pathol*, 2020 [37] Ren et al., *Front Genet*, 2021 [38] Zhang et al., *Front Mol Neurosci*, 2023 [18]
Slc20a2 knockout mice	Slc20a2	Slc20a2 gene knockout	Calcifications were found in the thalamus, hypothalamus, midbrain, pons, and cerebral cortex in homozygous 11-month-old mice.	Body weight and survival rate were decreased in homozygous mice.	Kurita et al., *Mol Brain*, 2025 [17]
Aldh1l1-CreERT2:Pit2^f/f^ mice	Slc20a2	Astrocyte-specific Slc20a2 knockout	Calcifications were found in the basal forebrain and hypothalamus in 12-month-old Aldh1l1-CreERT2:Pit2^f/f^ mice.	Pi concentration of CSF was increased in Aldh1l1-CreERT2:Pit2^f/f^ mice.	Cheng et al., *Neuron*, 2024 [19]
A humanized SLC20A2 intron mice (SLC20A2-KI)	Slc20a2	knocked in with the entire human intron 2 sequence (carrying the SLC20A2 c.289+1007 C>G variant)	Homozygous SLC20A2-KI mice began to show brain calcification in the hypothalamus and basal forebrain at the age of 5 months. Homozygous SLC20A2-KI mice exhibited calcification deposits widely distributed in the basal forebrain, thalamus, hypothalamus, midbrain, and pons at the age of 7 months.	Heterozygous and homozygous SLC20A2-KI mice exhibited significantly increased CSF Pi levels.	Zhao et al., *Neuron*, 2024 [39]
Disease-specific iPS cells for PBC	SLC20A2	SLC20A2 (c.1848C>G (p.Trp616Ter))	Not applicable	Decrease in Pi transport activity in endothelial cells derived from iPSC (Sekine et al., Biochem Biophys Res Commun, 2019 [42])	Sekine et al., *Stem Cell Res*, 2017 [43] Sekine et al., *Biochem Biophys Res Commun*, 2019 [42]
Disease-specific iPS cells for PBC	SLC20A2	SLC20A2 (c.613G>A (p.Val205Met))	Not applicable	Golgi damage (Sun et al., *Biochem Biophys Res Commun*, 2023 [44])	Zhang et al., *Stem Cell Res*, 2019 [45] Sun et al., *Biochem Biophys Res Commun*, 2023 [44]
Disease-specific iPS cells for PBC	SLC20A2	SLC20A2 (del exon10)	Not applicable	Golgi damage (Sun et al., *Biochem Biophys Res Commun*, 2023 [44])	Sun et al., *Biochem Biophys Res Commun*, 2023 [44]
Disease-specific iPS cells for PBC	SLC20A2	SLC20A2 (c.687dupT (p.Val230CysfsTer28))	Not applicable	No data	Begentas et al., *Stem Cell Res*, 2023 [46]
Pdgfb^ret/ret^ mice	Pdgfb	hypomorphic Pdgfb alleles	Calcifications were found in the basal forebrain, thalamus, midbrain and pons of homozygous 1-year-old mice (Keller et al., *Nat Genet*, 2013 [23]). Calcifications were found in the brains of homozygous 3-, 6-, and 12-month-old mice (Nahar et al., *Brain Pathol*, 2020 [37]).	Impairment of blood–brain barrier (BBB) permeability was observed in homozygous mice (Armulik et al., *Nature*, 2010 [25]; Nahar et al., *Brain Pathol*, 2020 [37]).	Keller et al., *Nat Genet*, 2013 [23] Armulik et al., *Nature*, 2010 [25] Nahar et al., *Brain Pathol*, 2020 [37]
Pdgfb^−/−^; R26P^+/0^ mice	Pdgfb	Pdgfb-null mice rescued to adulthood by transgenic re-expression of PDGF-B in the endothelium	Calcifications were found in the thalamus in 1-year-old Pdgfb^−/−^; R26P^+/0^ mice (Keller et al., *Nat Genet*, 2013 [23]).	Impairment of blood–brain barrier (BBB) permeability was observed in Pdgfb^−/−^; R26P^+/0^ mice (Armulik et al., *Nature*, 2010 [25]).	Keller et al., *Nat Genet*, 2013 [23] Armulik et al., *Nature*, 2010 [25]
Disease-specific iPS cells for PBC	PDGFB	PDGFB (c.160+2T>A)	Not applicable	Decrease in PDGFB level in the culture media from endothelial cells derived from iPSC (Sekine et al., *Sci Rep*, 2019 [47])	Sekine et al., *Sci Rep*, 2019 [47]
Disease-specific iPS cells for PBC	PDGFB	PDGFB (c.457-1G>T)	Not applicable	Decrease in PDGFB level in the culture media from endothelial cells derived from iPSC (Sekine et al., *Sci Rep*, 2019 [47])	Sekine et al., *Sci Rep*, 2019 [47]
Disease-specific iPS cells for PBC	PDGFB	PDGFB (c.33_34delCT)	Not applicable	Decrease in PDGFB level in the culture media from endothelial cells derived from iPSC (Sekine et al., *Sci Rep*, 2019 [47])	Sekine et al., *Sci Rep*, 2019 [47]
Xpr1 knockout mice	Xpr1	Xpr1 gene knockout	No data	Homozygous mice died, and showed placental calcification, embryonic calcification and growth restriction.	Xu et al., *J Bone Miner Res*, 2020 [40]
C57BL/6N-Xpr1^tm1a(KOMP)Wtsi^ mice (MGI: 4362650)	Xpr1	Xpr1 gene knockout	Calcifications were found in the thalamus of heterozygous 7-, 10-, 12-, and 16-month-old mice.	Pi concentration of CSF was decreased in heterozygous mice. Heterozygous mice present with an altered acoustic startle response.	Maheshwari et al., *Brain Pathol*, 2023 [41]
Myorg knockout mice	Myorg	Myorg gene knockout	Calcifications were found in the thalamus in homozygous 9-month-old mice (Yao et al., *Neuron*, 2018 [5]).	Pi concentration of CSF was increased in homozygous mice (Cheng et al., *Neuron*, 2024 [19]).	Yao et al., *Neuron*, 2018 [5] Cheng et al., *Neuron*, 2024 [19]
Jam2 knockout mice	Jam2	Jam2 gene knockout	No calcifications were found in brain.	Prominent vacuolation in the cerebral cortex, thalamus, and cerebellum and particularly widespread vacuolation in the midbrain were found in homozygous mice. Gait abnormalities were observed in homozygous mice.	Schottlaender ey al., *Am J Hum Genet*, 2020 [7]
Cmpk2 knockout mice	Cmpk2	Cmpk2 gene knockout	Calcifications were found in the thalamus of homozygous 10-, 12-, and 14-month-old mice.	Disruption of mitochondrial function was observed in homozygous mice.	Zhao et al., *Cell Discov*, 2022 [31]
Cmpk2 knock-in mice	Cmpk2	knocked in with c.2 T>C mutation	Calcifications were found in the thalamus of homozygous 12-month-old mice.	Disruption of mitochondrial function was observed in homozygous mice.	Zhao et al., *Cell Discov*, 2022 [31]
Disease-specific iPS cells for PBC	Not identified (from sporadic patient)	No data	Not applicable	No data	Yada et al., *Stem Cell Res*, 2021 [48]

### 3.2. In Vitro Models

Several PBC disease-specific iPSC have also been established (Table 2). Among these, there are numerous reports of iPSC established from *SLC20A2* mutant PBC patients [42,43,44,45,46]. Vascular endothelial cells differentiated from iPSC derived from these *SLC20A2* mutant PBC patients have been reported to exhibit reduced Pi uptake capacity [42] and Golgi apparatus dysfunction [44]. Additionally, it has been reported that PDGFB levels are low in the culture medium of vascular endothelial cells differentiated from iPSC established from *PDGFB*-mutated PBC patients [47]. Another iPSC line was also established from the sporadic PBC patient [48]. Thus, the number of disease-specific iPSC lines for PBC is limited, consisting almost exclusively of those established from patients with *SLC20A2*-mutant PBC and *PDGFB*-mutant PBC. Furthermore, phenotypic analysis remains underdeveloped. iPSC are important as human in vitro models. We believe that establishing iPSC with other PBC familial causative gene mutations and conducting detailed phenotypic analyses of their differentiation into nervous system cells (neurons, astrocytes, microglia, vascular cells, pericytes, vascular smooth muscle cells, and vascular endothelial cells) would be essential for studying the disease mechanism and developing therapeutic drugs.

## 4. Discussion

The frequency of PBC in the population is estimated about 2.1–6.6 per 1000 people [49,50]. PBC had been considered a rare disease for a long time [51]. Therefore, there is difficulty for PBC in funding the research and developing new therapeutic options due to socio-economical aspects. Although the pathogenesis and treatment of PBC remain unclear, analysis of several familial causative genes and genetically modified mice targeting these genes has gradually elucidated its underlying mechanisms. Dysfunction of the PBC causative genes *SLC20A2* and *XPR1* is thought to be a cause of abnormal Pi metabolism in cells. SLC20A2 and XPR1 interact with each other, and PP-IP derived from IP6K taken up by SLC20A2 is required for Pi excretion by XPR1 [29]. Pi levels are elevated in the CSF of *Slc20a2* homozygous KO mice and *SLC20A2* mutant PBC patients [21,22], whereas Pi levels are low in the CSF of *Xpr1* heterozygous KO mice [41]. Therefore, it is considered that intracellular and extracellular Pi homeostasis cannot be maintained unless both SLC20A2 and XPR1 function properly. Furthermore, analysis of other familial PBC causative genes—*PDGFB*, *PDGFRB*, and *JAM2*—along with studies using astrocyte-specific *Slc20a2* KO mice, suggests that disruption of the neurovascular unit (NVU), such as increased BBB permeability, may also contribute to intracerebral calcification [3,19,23,30]. On the other hand, MYORG, CMPK2, and NAA60 are thought to be involved in PBC pathogenesis through some form of target protein modification or nucleic acid metabolism mechanisms. Some reports indicate that MYORG is involved in the distribution of Pit2 to the cell membrane in astrocytes [19], and that NAA60 could acetylate the N-terminus of the Pit2 protein to regulate its expression at the cell membrane [33]. Detailed molecular mechanism analyses using genetically modified mice targeting these PBC causative genes are expected to lead to the discovery of therapeutic targets for PBC.

**Figure 1:**
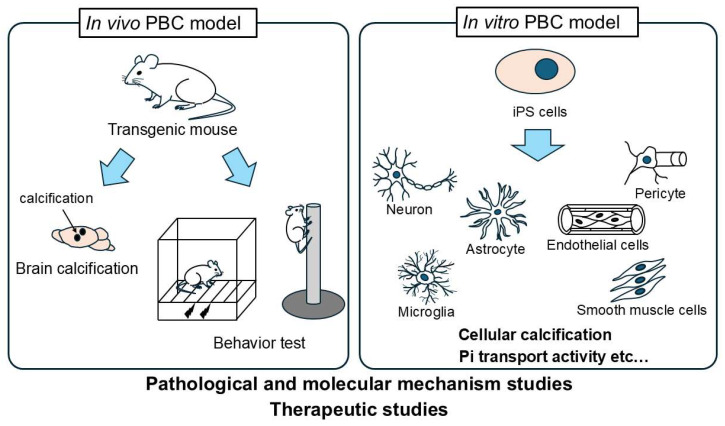
Importance of in vitro and in vivo PBC models. PBC models are crucial for evaluating candidate therapeutics in PBC drug discovery research. Generally, a combination of in vivo and in vitro evaluation systems is required. In vivo PBC models should be able to reproduce neurobehavioral phenotypes such as intracerebral calcification, abnormalities in Pi metabolism, and higher brain functions. We consider experimental systems using iPSC to be useful for in vitro drug evaluation systems. iPSC is important as an appropriate human experimental evaluation system, and it is considered that they should be used to complement in vivo models. It is considered necessary to develop in vitro drug evaluation systems using various cells constituting the NVU differentiated from iPSC, such as those that evaluate phosphate uptake capacity and cellular calcification.

On the other hand, PBC models are crucial for evaluating candidate therapeutics in PBC drug discovery research. When comparing in vivo and in vitro PBC models, the advantage of the in vivo PBC mouse model is its ability to reproduce phenotypes observed in PBC patients, such as intracerebral calcification and higher brain functions. The disadvantage of in vivo PBC models is that they are costly and time-consuming for drug evaluation. The advantages of using an in vitro iPSC PBC model include the ability to utilize human cells, allowing consideration of species differences, and its suitability for drug screening compared to in vivo approaches. The disadvantages of in vitro PBC models include the limited types of iPS cells that can be used, and the difficulty in reproducing calcification. Therefore, a combination of in vivo and in vitro evaluation systems is required (Figure 1). In vivo PBC models should be able to reproduce neurobehavioral phenotypes such as intracerebral calcification, abnormalities in Pi metabolism, and higher brain functions. From that perspective, while the *Slc20a2* KO mouse currently meets these criteria, considering the diverse pathologies of PBC, evaluation using a single PBC model is insufficient. Therefore, it is ideal to establish and utilize other types of KO mice for familial PBC causative genes and knock-in mice for human PBC mutations as PBC models for evaluation. It is considered necessary to establish in vivo PBC models targeting other PBC familial genes besides *SLC20A2*. We consider experimental systems using iPSC to be useful for in vitro drug evaluation systems; however, for PBC, the number of cell types is limited, and phenotypic information is also scarce. iPSC is important as an appropriate human experimental evaluation system, and it is considered that they should be used to complement in vivo models. There have been no reports of reproducing calcification using PBC-derived iPSC or cells differentiated from iPSC. It is considered necessary to develop in vitro drug evaluation systems using various cells constituting the NVU differentiated from iPSC, establish 3D cultures, co-cultures, and organoids using these cells, and evaluate Pi uptake capacity and cellular calcification in these models.

Therefore, while it is important to utilize both in vitro and in vivo PBC model systems in drug discovery research and studies of the disease mechanism, this approach is still considered to be in its developmental stage. Furthermore, while no PBC models have been reported to date, utilizing models based on nematodes, Drosophila, or AI tools for anticipating therapeutic molecular targets and iPSC-derived organoids could also prove useful. We anticipate that further functional analysis of genes responsible for familial cases of the disease will clarify their involvement in its pathogenesis, leading to the development of improved models of PBC.

## Figures and Tables

**Table 1:** Summary of PBC familial causative gene.

Gene Name	Functions	Mutations Involved [10]	Frequency in Genetic-Confirmed PBC Patients [10]
SLC20A2	phosphate transporter	Single-nucleotide variants (SNVs) (Most common variants were missense.), structural variants, and in-frame insertions/deletions	58%
PDGFB	formation of BBB	SNVs (Most common variants were missense.), structural variants and small frameshift insertions or deletions	13%
PDGFRB	formation of BBB	SNVs (Only missense)	4%
XPR1	phosphate transporter	SNVs (Most common variants were missense.)	6%
MYORG	others/unknown	SNVs (Most common variants were missense.), and small insertions or deletions including in-frame deletions	14%
JAM2	formation of BBB	SNVs (Most common variants were nonsense.), small frameshift insertions/deletions and structural or splice site/region variants	3%
CMPK2	mitochondrial function	SNVs (Most common variants were missense and start codon loss.)	unknown
NAA60	others/unknown	SNVs (Most common variants were missense.), and a smaller fraction are frameshift deletions, insertions, or splice site region variants.	2%

## Data Availability

No new data were created or analyzed in this study.
